# Effect of Annealing Temperature on the Water Contact Angle of PVD Hard Coatings

**DOI:** 10.3390/ma6083373

**Published:** 2013-08-07

**Authors:** Yu-Sen Yang, Ting-Pin Cho

**Affiliations:** 1 Department of Mechanical and Automation Engineering, National Kaohsiung First University of Science and Technology, 2 Juoyue Rd., Nantz District, Kaohsiung 81164, Taiwan; 2 Institute of Engineering Science and Technology, National Kaohsiung First University of Science and Technology, 2 Juoyue Rd., Nantz District, Kaohsiung 81164, Taiwan; E-Mail: tpcho@mail.mirdc.org.tw; 3 Metal Industries Research & Development Centre, 1001 Kaonan Highway, Kaohsiung 811, Taiwan

**Keywords:** nitride, DLC, hydrophobic, hydrophilic, water contact angle, anti-sticking

## Abstract

Various PVD (physical vapor deposition) hard coatings including nitrides and metal-doped diamond-like carbons (Me-DLC) were applied in plastic injection and die-casting molds to improve wear resistance and reduce sticking. In this study, nitrides hcp-AlN (hexagonal close-packed AlN), Cr_2_N, (CrAl)_2_N) and Me-DLC (Si-DLC and Cr-DLC) coatings were prepared using a closed field unbalanced magnetron reactive sputtering system. The coatings were annealed in air for 2 h at various temperatures, after which the anti-sticking properties were assessed using water contact angle (WCA) measurements. The as-deposited hcp-AlN, Cr_2_N and (CrAl)_2_N coatings exhibit hydrophobic behavior and exhibit respective WCAs of 119°, 106° and 101°. The as-deposited Si-DLC and Cr-DLC coatings exhibit hydrophilic behavior and exhibit respective WCAs of 74° and 88°. The annealed Cr_2_N and (CrAl)_2_N coatings exhibit hydrophobic behavior with higher WCAs, while the annealed hcp-AlN, Si-DLC and Cr-DLC coatings are hydrophilic. The increased WCA of the annealed Cr_2_N and (CrAl)_2_N coatings is related to their crystal structure and increased roughness. The decreased WCA of the annealed hcp-AlN, Si-DLC and Cr-DLC coatings is related to their crystal structures and has little correlation with roughness.

## 1. Introduction

Physical vapor deposition (PVD) processes have long been widely used in the preparation of hard coatings due to their superior combined properties, such as high hardness and good resistance to wear, corrosion and oxidation [1,2,3,4,5]. In addition to wear resistance, corrosive resistance and thermal stability, anti-sticking is also an important property for plastic injection molds [6,7] and the anti-sticking property of coatings affects the release performance of plastic injection molds.

To provide acceptable results, hard coatings must be selected carefully for use in plastic injection molds [6], and good release performance requires low surface energy [6,7]. The coating’s anti-sticking property is related to the polar components of surface energy, with lower polar components corresponding with increased water-repellency as well as higher water contact angle (WCA) [7]. A surface with a WCA for water below 90° can be called a hydrophilic surface, while surfaces with a WCA greater than 90° are hydrophobic [8]. A higher WCA corresponds with improved release performance for plastic injection molds.

PVD nitride and diamond-like carbon (DLC) coatings exhibit good wear and corrosion resistance [9,10,11,12]. In particular, the DLC coating exhibits a lower friction coefficient [13,14,15,16]. Previous studies have indicated that the anti-sticking property of the Cr–N coating outperforms that of the Zr-DLC coating in practical injection process [17].

In this study, hcp-AlN, Cr_2_N, (CrAl)_2_N, Si-DLC and Cr-DLC coatings were prepared using a closed field unbalanced magnetron (CFUBM) reactive sputtering system and further annealed in air for 2 h at various temperatures. The hydrophobic and hydrophilic properties of as-deposited and annealed coatings were investigated and the relationship between the phase structures and their respective WCAs was also discussed.

## 2. Experimental Details

### 2.1. Coatings Preparation

**Figure 1:**
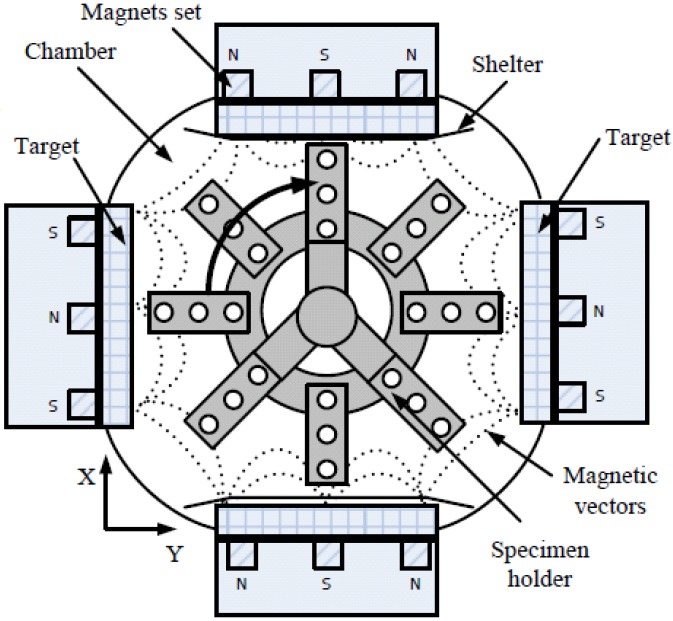
Schema of the closed field unbalanced magnetron reactive sputtering system.

The AlN, Cr_2_N, (CrAl)_2_N, Si-DLC and Cr-DLC coatings were prepared using a CFUBM reactive sputtering system with four vertical cathodes at intervals of 90°. The Schema of the CFUBM reactive sputtering system is shown in Figure 1. Dimensions of the deposition chamber were 550 mm in diameter and 500 mm in height, while the dimensions of the target are 300 mm × 109 mm × 10 mm.

Two face-to-face Al targets were powered by a medium-frequency (MF) power source for the deposition of the AlN coating. Two face-to-face Cr targets were powered by a DC (Direct Current) power source for the deposition of the Cr_2_N coating. For the deposition of the (CrAl)_2_N coating, two face-to-face Cr targets were powered by a DC power source. The other Al and Cr targets were powered by a MF (Medium-frequency, 40 kHz) power source. The Si-DLC coating was deposited with a Si target and three C targets. Two face-to-face C targets were powered by a DC power source. The other C and Si targets were powered by a MF power source. The Cr-DLC was deposited with two face-to-face Cr targets. The respective purity of the Cr, Al, Si and C target materials was 99.5%, 99.999%, 99.999% and 99.98%. The gas purity of Ar, N_2_ and C_2_H_2_ was 99.999%. The substrate bias was powered using pulsed DC with a pulse width of 1056 ns.

Morphological anisotropy will affect the water contact angle [18] and this impact was minimized in this study by using polished Si (100) substrates. Si (100) substrates were cleaned in an ultrasonic cleaner with surfactant for 15 min and then with de-ionized water for 10 min, and then dried at 100 °C for 15 min before the coating deposition. Prior to deposition, the coating chamber was pumped down to 2.6 × 10^−3^ Pa. Substrates were bombarded using argon ion (Ar^+^) at a pressure 0.57 Pa and a bias of −450 V for 10 min before deposition. The thickness of all specimens was controlled at 2.0 ± 0.1 μm by controlling the deposition time. The deposition temperature of all coatings is about 200 ± 20 °C. Previous studies have shown that the N_2_ partial pressure affects the crystal structures of Cr–N [10]. In this study, the N_2_ partial pressure was controlled to prepare the Cr_2_N and (CrAl)_2_N coatings. All of the deposition parameters are shown in Table 1.

**Table 1:** Deposition parameters for various coatings.

Coatings	AlN	Cr_2_N	(CrAl)_2_N	Si-DLC	Cr-DLC
Flow rate Ar/N_2_/C_2_H_2_ (sccm)	30/12/0	34/16/0	30/20/0	35/0/0	25/5/20
Working pressure (Pa)	0.40	0.41	0.47	0.38	0.43
Target materials	Al × 2	Cr × 2	Cr × 3 + Al × 1	C × 3 + Si × 1	Cr × 2
Target current (A)	Al 6A	Cr 5A	Cr 4A + (Cr + Al) 1A	C 3A + (C + Si) 1.5A	Cr 5A
Substrate Bias Frequency (kHz)	40	130	150	100	70
Negative bias of substrate (−V)	100	50	100	60	75
Rotation speed of specimen (rpm)	5	9	7	9	9
Distance, target to specimen (cm)	7	11	11	9	13
Deposition time (min)	420	25	60	150	80
As-deposited roughness (nm)	54	4	13	3	6
Annealing temperature (°C)	300, 600–800	300–800	300, 600–800	200–600	200, 300

### 2.2. Post Annealing of Coatings

**Figure 2:**
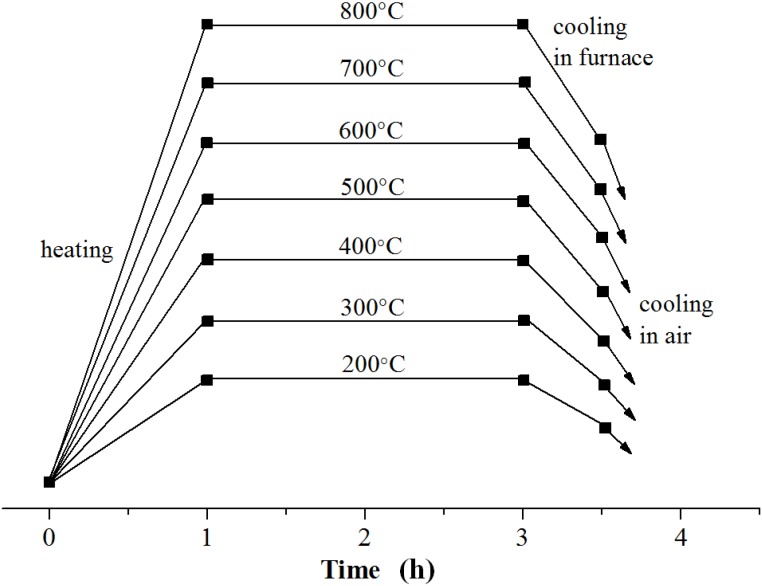
Coating annealing process.

The oxidation resistant temperatures of the Cr_2_N, (CrAl)_2_N and DLC are around 800, 900 and 400 °C, respectively [3,19,20]. The process of the annealing is shown in Figure 2. All of the coatings were annealed in air for 2 h at various fixed temperatures to investigate the effect of annealing temperature on the surface morphologies, crystal structures and water contact angle (WCA) of the coatings.

### 2.3. Characterization

**Figure 3:**
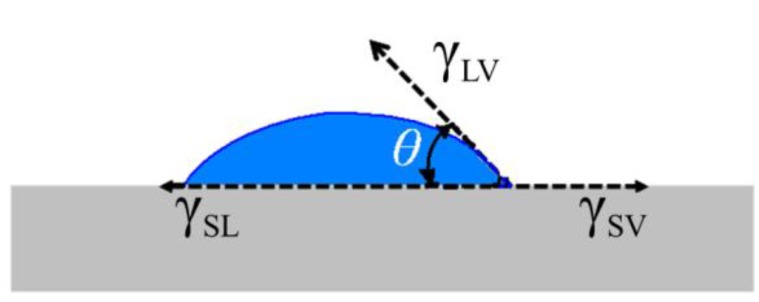
Diagrammatic sketch of water contact angle *θ*.

The crystal structure of the coatings was characterized by grazing incidence X-ray diffractometer (D1 HR-XRD, Bede, Durham, UK) with Cu Kα radiation (λ = 1.5418 Å, 2*θ*/min = 3°). The crystal structures of the annealed AlN, (CrAl)_2_N and Si-DLC films were also observed using X-ray Photoelectron Spectrometer (XPS, PHI-5000 VersaProbe, PHI, Chanhassen, MN, USA). The thickness and morphologies of the various coatings were observed by field emission scanning electron microscopy (FE-SEM, Hitachi-4700, Hitachi, Tokyo, Japan) with an accelerating voltage of 15 kV. The Al content of the (CrAl)_2_N films was measured using energy dispersive X-ray spectroscopy (EDS) analysis in the Hitachi-4700 FE-SEM. The crystal structure of the as-deposited Si-DLC was characterized using a micro-Raman system (HR-800, HORIBA, Kyoto, Japan) at a backscattering configuration with a 100× optical microscope objective. The surface roughness Ra of the coatings was measured by a surface profiler (Alpha-Step IQ, KLA-Tencor, Milpitas, CA, USA) with a vertical resolution of 0.24 Å. The measurement distance and scanning speed were 800 μm and 50 μm/s, respectively. The sessile-drop method was used for the WCA measurement using a contact angle measurement device (FTA-200, First Ten Angstroms, Portsmouth, UK). Figure 3 shows a diagrammatic sketch of the water contact angle *θ*.

The equilibrium of forces among the surface tensions at the 3-phase boundary is described by Young’s Equation [21].

(1) $$\gamma_{SV}=\gamma_{SL}+\gamma_{LV}\cos\theta$$

where $\gamma_{SV}$, $\gamma_{SL}$ and $\gamma_{LV}$ are respectively the surface energy of the solid–vapor, solid–liquid and liquid–vapor interfaces, and *θ* is the equilibrium contact angle.

## 3. Results and Discussion

**Figure 4:**
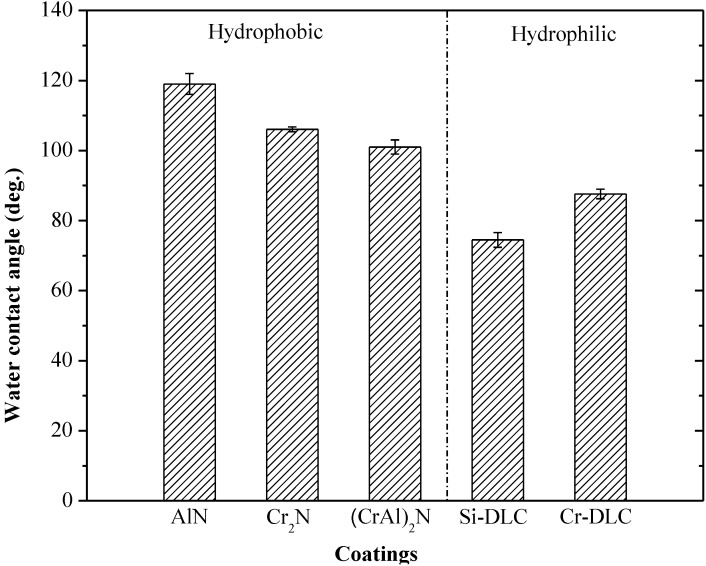
Water contact angle (WCA) of as-deposited coatings.

The WCA of the as-deposited AlN, Cr_2_N, (CrAl)_2_N, Si-DLC and Cr-DLC coatings are shown in Figure 4. The as-deposited AlN, Cr_2_N and (CrAl)_2_N coatings exhibit hydrophobic behavior and have respective WCAs of 119°, 106° and 101°. The as-deposited Si-DLC and Cr-DLC coatings exhibit hydrophilic behavior and have respective WCAs of 74° and 88°.

**Figure 5:**
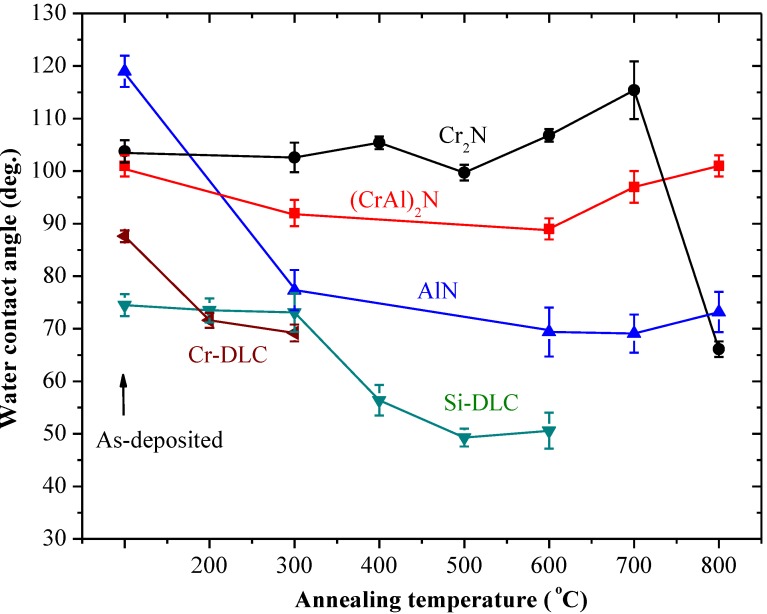
WCA of the AlN, Cr_2_N, (CrAl)_2_N, Si-DLC and Cr-DLC coatings annealed at various temperatures.

Figure 5 shows the WCAs of the annealed AlN, Cr_2_N, (CrAl)_2_N, Si-DLC and Cr-DLC coatings. The WCA of the 300 °C annealed AlN sharply decreases to 78°. The AlN annealed at higher temperatures still has a lower WCA of 70°~74°. For annealing temperature below 500 °C, the WCA of the Cr_2_N coating shows no noticeable change and still greater than 100°. As the annealing temperature rises to 700 °C, the WCA increases to 115°. The WCA of the 800 °C annealed Cr_2_N coating rapidly drops to 66°. As the annealing temperature rises to 600 °C, the WCA of the (CrAl)_2_N decreases to 90°. However, the WCA increases to 101° as the annealing temperature rises to 800 °C. The WCAs of the annealed Si-DLC and Cr-DLC coatings are lower than those of the as-deposited and still show hydrophobic behavior. With increased carbon content, the Cr-DLC film is damaged after annealing higher than 400 °C. Annealing of the Cr-DLC film was kept below 300 °C and the WCA of the annealed Cr-DLC decreased to about 70°. The WCA of the annealed Si-DLC decreases to 50° as annealing temperature rises to 500 °C. Both the annealed Si-DLC and Cr-DLC are hydrophilic.

**Figure 6:**
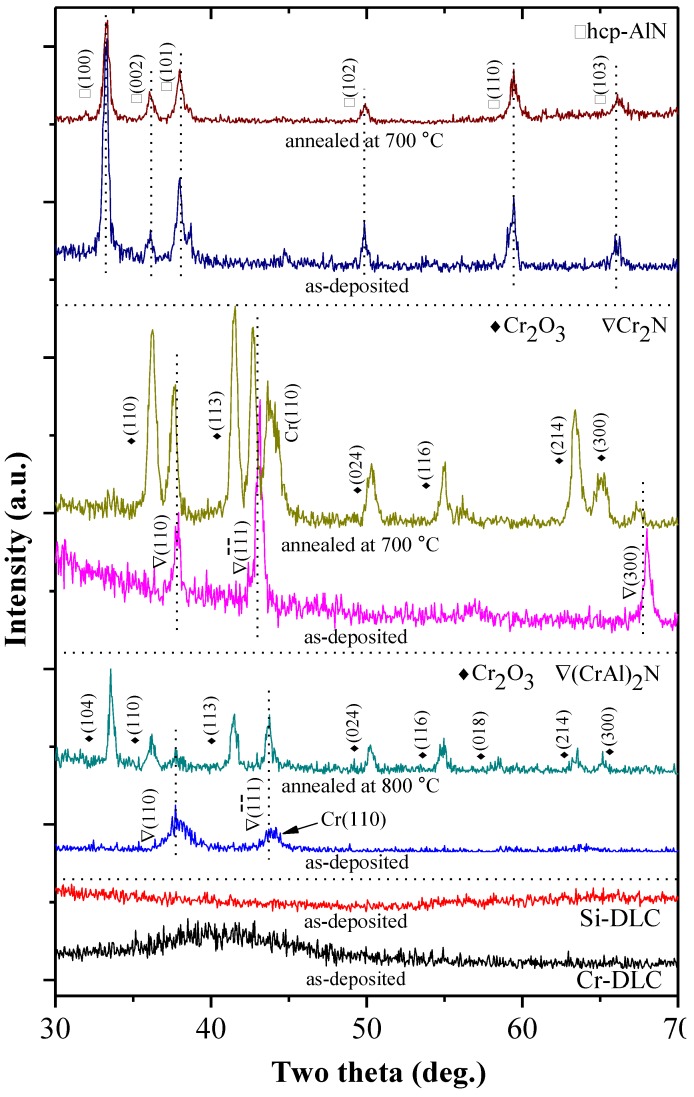
XRD patterns of various coatings.

The WCAs of the coatings are related to the crystal structure and surface roughness [7,22]. X-ray diffraction (XRD) patterns were generated to investigate the crystal structure and oxidation behavior of the coatings. Figure 6 shows the XRD patterns of the as-deposited and selected annealed AlN, Cr_2_N, (CrAl)_2_N, Si-DLC and Cr-DLC coatings. The XRD pattern of the as-deposited AlN coating exhibits a hexagonal close-packed (hcp) structure and prefers an orientation of (100). As the hcp-AlN coating annealed at 700 °C, the intensity of plane (100) decreases significantly and the peaks of the oxide Al_2_O_3_ phase cannot be clearly identified. The XRD pattern of the as-deposited Cr_2_N coating shows a hexagonal structure and prefers an orientation of ($\bar{1}\bar{1}1$). The Cr_2_O_3_ peaks can be clearly identified after annealing at 700 °C. The decomposition of Cr_2_N and formation of Cr_2_O_3_ can be expressed as [20]:

Cr_2_N → 2Cr + N_2_ (2)

4Cr + 3O_2_ → 2Cr_2_O_3_ (3)

According to the EDS analysis results, the Al content of the (CrAl)_2_N coating is only 1.6%. The XRD pattern of the as-deposited (CrAl)_2_N features only two peaks at (110) and ($\bar{1}\bar{1}1$), similar to the pattern for the Cr_2_N coating. The Cr_2_O_3_ peaks of the 700 °C annealed (CrAl)_2_N coating can be clearly identified. There are no obvious XRD peaks for the as-deposited Si-DLC and Cr-DLC. As shown in Figure 6, the broadening of the peak for Cr-DLC would result from the presence of ultra fine grain carbide in the carbon matrix [14].

**Figure 7:**
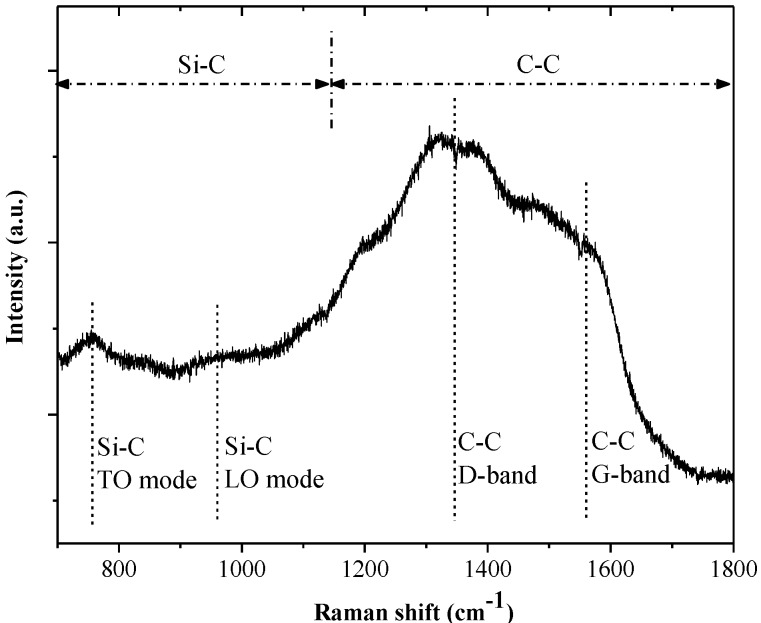
Raman spectrum of Si-DLC.

The Si-containing DLCs are amorphous structures. Figure 7 shows the Raman spectrum of the as-deposited Si-DLC coating for further structural examination. The dotted lines on the top of Figure 7 roughly indicate two regions corresponding to the different Si–C and C–C vibration modes [23,24]. Two broad bands centered at ~766 and ~966 cm^−^^1^ are SiC Raman scattering peaks for the TO (transverse optical) and LO (longitudinal optical) modes [24]. The D band (centered near 1350–1400 cm^−1^) and G band (centered near 1560 cm^−1^) are caused by the C–C vibration mode [24]. The G peak is due to the bond stretching of all pairs of sp^2^ atoms in both rings and chains. The D peak is due to the breathing modes of the rings. A peak centered at ~1560 cm^−1^ in the Raman spectrum is characteristic of DLC coatings, and is also described as the “fingerprint” of the DLC coating [14,15].

**Figure 8:**
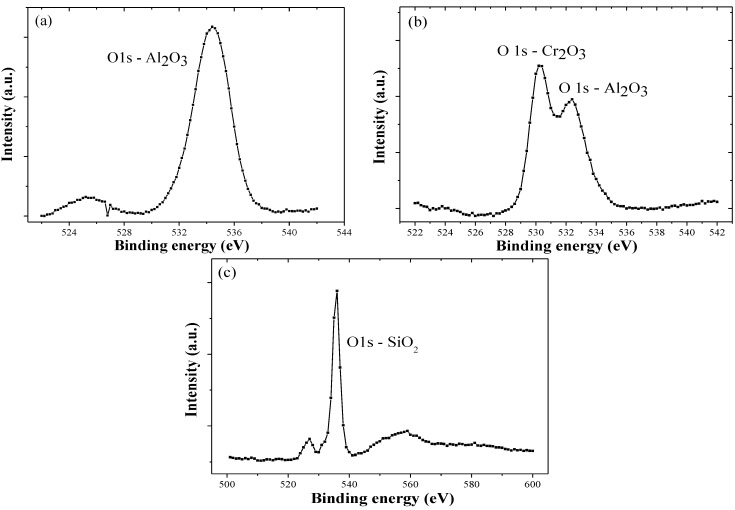
XPS spectrum of (**a**) hcp-AlN annealed at 300 °C; (**b**) 600 °C annealed (CrAl)_2_N and (**c**) 400 °C annealed Si-DLC.

Figure 8 shows the XPS spectra of 300 °C annealed hcp-AlN, 600 °C annealed (CrAl)_2_N and 400 °C annealed Si-DLC coatings. The Al_2_O_3_ phase of the 300 °C annealed hcp-AlN and 600 °C annealed (CrAl)_2_N coatings are clearly identified using the XPS spectra measurement. The oxidation of the retained A1 atoms resulted in the formation of a thin Al_2_O_3_ film on the surface of the annealed hcp-AlN and (CrAl)_2_N coatings. Figure 8c shows the XPS spectrum of the 400 °C annealed Si-DLC coating, and indicates that the oxidation of the retained Si atoms causes the formation of the SiO_2_ phase in the Si-DLC coatings.

In addition to the chemical composition of the material surface, the hydrophobic or hydrophilic behavior of the coatings can be enhanced by the degree of surface roughness [8]. Wenzel proposed the following equation for liquid wetting in a rough, chemically homogeneous substrate [22]:

cos θ* = R·cos *θ* (4)

where *θ* is the intrinsic contact angle on the microscopic flat surface, θ* is the contact angle on a rough surface and R is the ratio of the actual area of the rough surface to the geometric projected area (R ≥ 1). Wenzel’s equation indicates that the effect of the surface roughness strongly depends on the value of the intrinsic contact angle *θ* [25,26].

Since R ≥ 1, rougher surface make hydrophilic surfaces more hydrophilic (θ* < *θ*) and hydrophobic surfaces more hydrophobic (θ* > *θ*).

**Figure 9:**
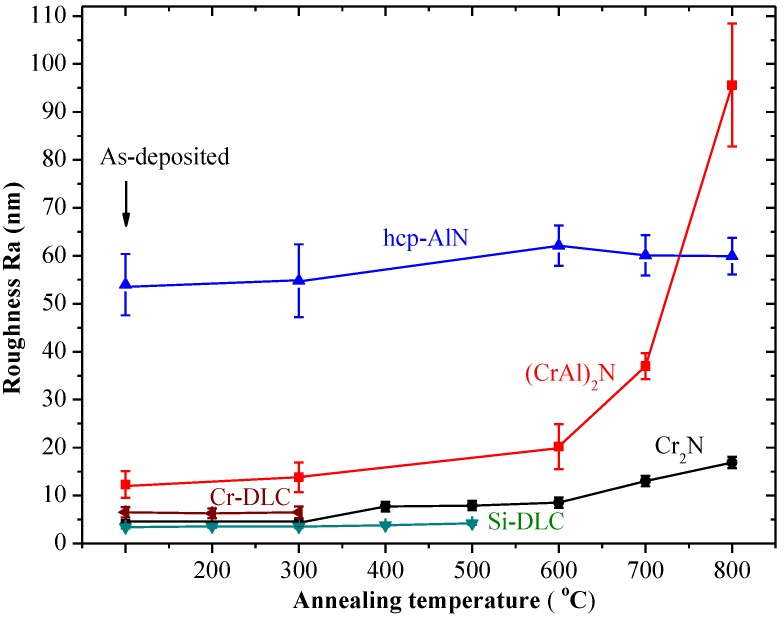
Roughness Ra of the as-deposited and annealed at various temperatures for the hcp-AlN, Cr_2_N, (CrAl)_2_N, Si-DLC and Cr-DLC coatings.

Figure 9 shows the roughness Ra of the as-deposited and annealed hcp-AlN, Cr_2_N, (CrAl)_2_N, Si-DLC and Cr-DLC coatings. There is no obvious difference of roughness between the as-deposited and the annealed coatings for the hcp-AlN, Si-DLC and Cr-DLC. The roughness of the 700 °C and 800 °C annealed Cr_2_N coatings respectively increases to 13 nm and 17 nm. The roughness of the 800 °C annealed (CrAl)_2_N coatings significantly increases to 96 nm.

**Figure 10:**
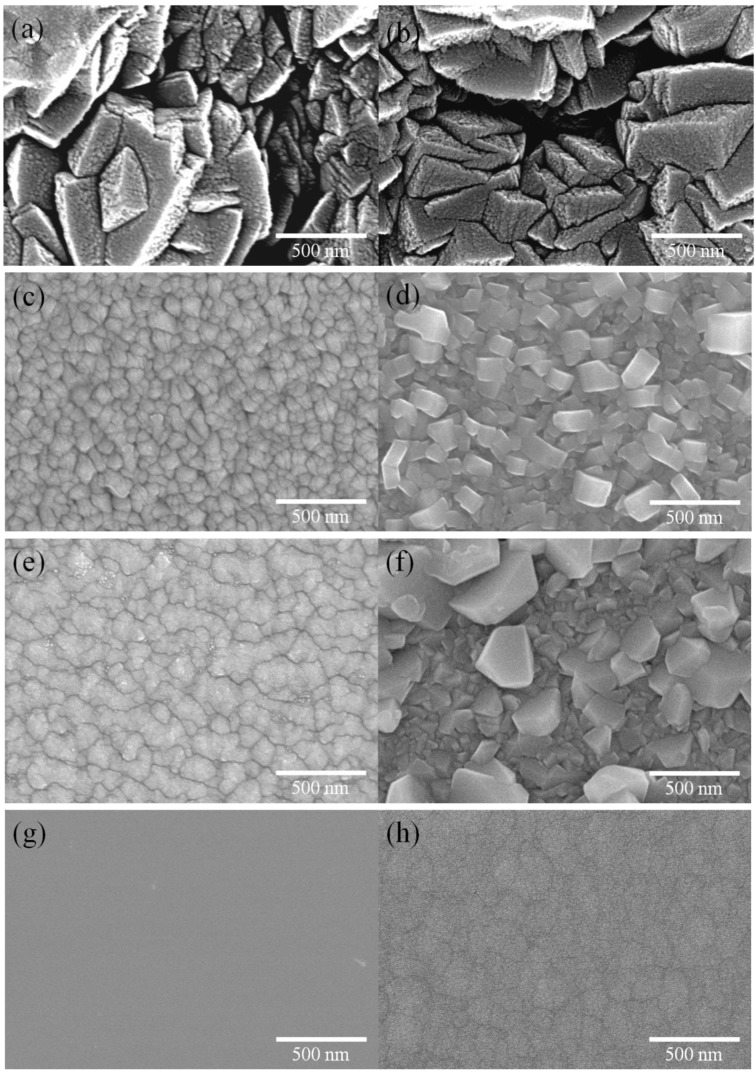
SEM images (**a**) as-deposited hcp-AlN; (**b**) 600 °C annealed hcp-AlN; (**c**) as-deposited Cr_2_N; (**d**) 600 °C annealed Cr_2_N; (**e**) as-deposited (CrAl)_2_N; (**f**) 700 °C annealed (CrAl)_2_N; (**g**) as-deposited Si-DLC; and (**h**) as-deposited Cr-DLC.

Figure 10 shows the surface morphologies observed by SEM for the hcp-AlN, Cr_2_N, (CrAl)_2_N, Si-DLC and Cr-DLC coatings. Figure 10a, c, e, g and h, respectively show the as-deposited hcp-AlN, Cr_2_N, (CrAl)_2_N, Si-DLC and Cr-DLC coatings. Figure 10b shows the hcp-AlN coating annealed at 600 °C. No oxidation is apparent, and the morphology is similar to that of the as-deposited hcp-AlN coating shown in Figure 10a. Figure 10d presents the morphology of the Cr_2_N coating annealed at 600 °C, showing that the surface is covered by the rough crystallite of Cr_2_O_3_. Figure 10f shows the morphology of the (CrAl)_2_N coating annealed at 700 °C. It differs from the as-deposited (CrAl)_2_N coating, with small oxide grains of Cr_2_O_3_ forming on the surface. According to the results shown in Figure 6, Figure 9 and Figure 10, the rougher surface of the annealed Cr_2_N and (CrAl)_2_N is due to the formation and growth of the Cr_2_O_3_ crystals on the coating’s surface. Figure 10g and h, respectively present the morphologies of the Si-DLC and Cr-DLC coatings, showing smoother surfaces than those found in the hcp-AlN and (CrAl)_2_N coatings.

The as-deposited hcp-AlN coating possesses the highest WCA of 119°. As shown in Figure 5, the WCA of the 300 °C annealed hcp-AlN coating sharply decreases to 78°. As shown in Figure 6, there is no obvious difference between the XRD patterns of the hcp-AlN coatings either as-deposited or annealed at 700 °C. In Figure 8a, the Al_2_O_3_ phase of the 300 °C annealed hcp-AlN coating is clearly identified using the XPS spectra measurement. Al_2_O_3_ films possess hydrophilic characteristics [27], which imply that the lower WCAs of the annealed hcp-AlN coatings are related to the Al_2_O_3_ thin film on the coating. As shown in Figure 9, there is no obvious difference in roughness between the as-deposited and annealed hcp-AlN coatings, indicating that the WCA of the annealed hcp-AlN is related to the crystal structure and has little correlation with roughness.

According to Figure 5 and Figure 9, the WCA and roughness of the as-deposited Cr_2_N coating are about 104° and 4 nm, respectively. As shown in Figure 5, for annealing temperatures below 500 °C, there is no obvious change to the WCA which remains above 100°. As the annealing temperature rises to 700 °C, the WCA increases to 115° with a rougher surface (Ra = 13 nm). According to Figure 5 and Figure 9, the increase to WCA results from a combination of retained Cr_2_N and higher roughness. As shown in Figure 9, the 800 °C annealed Cr_2_N coating exhibits a rougher surface with a roughness of 17 nm. As Cr_2_O_3_ is a hydrophilic material [27], the rougher surface causes the WCA of the 800 °C annealed Cr_2_N coating to rapidly drop to 66°. The WCA and roughness of the as-deposited (CrAl)_2_N coating are about 101° and 12 nm, respectively. The XPS spectrum of the 600 °C annealed (CrAl)_2_N coating is shown in Figure 8b. According to the XRD pattern and the XPS spectrum, both Al_2_O_3_ and Cr_2_O_3_ are formed on the surface of the 600 °C annealed (CrAl)_2_N coating and the roughness Ra slightly increasing to 20 nm. As both Al_2_O_3_ and Cr_2_O_3_ are hydrophilic films [27], the WCA slightly decreases to 90°. As the annealing temperature rises to 800 °C, the WCA increases to 101° given retained (CrAl)_2_N and a rougher surface (Ra = 106 nm). The WCAs of the annealed Cr_2_N and (CrAl)_2_N coatings are related to the crystal structure and increased roughness.

Figure 8c shows the XPS spectrum of the 400 °C annealed Si-DLC coating, indicating that the SiO_2_ phase is formed in the Si-DLC coatings due to the oxidation of the Si atoms. SiO_2 _shows hydrophilic behavior [28]. The WCA of the 400 °C annealed Si-DLC decreases to 56°. With higher carbon content, the Cr-DLC coating is damaged after annealing at 400 °C or higher. The WCA of the 300 °C annealed Cr-DLC also decreases to about 70°. Both the annealed Si-DLC and Cr-DLC are hydrophilic. According to Figure 9, the roughness of both the as-deposited and annealed Si-DLC and Cr-DLC coatings are stable. This indicates the WCAs of the annealed Si-DLC and Cr-DLC coatings are related to the crystal structure and has little correlation with roughness.

## 4. Conclusions

Various PVD hard coatings including nitrides and metal-doped diamond-like carbons (Me-DLC) were applied to plastic injection or die-casting molds to increase wear resistance and reduce sticking. In this study, hcp-AlN, Cr_2_N, (CrAl)_2_N), Si-DLC and Cr-DLC coatings were prepared using a closed field unbalanced magnetron reactive sputtering system. The WCA of the as-deposited and the annealed coatings were measured. Higher degrees of WCA corresponded with increased water-repellency as well as better anti-sticking properties. The research results for the various coatings can be summarized as:

1. The as-deposited hcp-AlN, Cr_2_N and (CrAl)_2_N coatings exhibit hydrophobic behavior and respectively possess WCAs of 119°, 106° and 101°. On the contrary, the as-deposited Si-DLC and Cr-DLC coatings exhibit hydrophilic behavior and respectively possess WCAs of 74° and 88°.
2. The annealed Cr_2_N and (CrAl)_2_N coatings are characterized by hydrophobic behavior with higher degrees of WCA. The annealed hcp-AlN coating is characterized by hydrophilic behavior and differs from the as-deposited one. Both the annealed Si-DLC and Cr-DLC are hydrophilic.
3. The annealed Cr_2_N coating exhibits the best anti-sticking property with the highest WCA. As the annealing temperature rises to 700 °C, the WCA increases to 115°. The increase of WCA is the result of the retained Cr_2_N and the increased roughness. According to the roughness results as shown by SEM and XRD, the rougher surface of the annealed Cr_2_N and (CrAl)_2_N is due to the formation and growth of Cr_2_O_3_ crystals on the coating’s surface.
4. For the 600 °C annealed (CrAl)_2_N coating, the WCA decreases to 90° and the roughness only slightly increases to 20 nm. The decrease of the WCA is caused by the formation of the Cr_2_O_3_ and Al_2_O_3_ on the coating surface. The WCA of the 800 °C annealed (CrAl)_2_N coating has a WCA of 101° with retained (CrAl)_2_N and a rougher surface. The increase in WCA is caused by the retained (CrAl)_2_N and increased roughness.
5. There is no obvious difference of roughness between the as-deposited and the annealed hcp-AlN coatings. The lower WCA of the annealed hcp-AlN coatings is related to the Al_2_O_3_ thin film on the coating, meaning that the WCA of the annealed hcp-AlN is related to the crystal structure and has little correlation with roughness.
6. The roughness of both the as-deposited and annealed Si-DLC and Cr-DLC coatings is stable. This indicates that the WCAs of the annealed Si-DLC and Cr-DLC coatings are related to the crystal structure and has little correlation with roughness.
